# Histochemical analysis and storage behaviour of Ginger (*Zingiber officinale* Roscoe) under Zero-Energy Cool Chamber (ZECC)

**DOI:** 10.1371/journal.pone.0265320

**Published:** 2022-05-05

**Authors:** Dharini Chittaragi, Jalaja S. Menon, Anoop E. V.

**Affiliations:** 1 Department of Spices and Plantation Crops, Tamil Nadu Agricultural University, Coimbatore, India; 2 Cashew Research Station, Kerala Agricultural University, Thrissur, India; 3 College of Forestry, Kerala Agricultural University, Thrissur, India; Mohanlal Sukhadia University, INDIA

## Abstract

In Kerala, a coastal land in India, Ginger is cultivated as a rainfed annual. The current study on morphological characters of seed rhizomes stored in Zero Energy Cool Chambers recorded a weight loss of 28% at three months after storage. The number of sprouting buds was maximum (12.25) in the seed rhizomes stored for three months. The dimensions of the bud measured at the varied periods of storage interval showed variation. The length of the bud increased from 0.847μm to 2.19 μm and the breadth reduced from 1.19 μm to 0.703μm in three months of storage. The current study provides the anatomical morphology of ginger seed rhizomes. Histochemical studies of seed rhizome for three months storage showed that the number of cork layers varied from 5–15, size of starch grain decreased on storage from 40 μm to 20 μm and the oil globules found inside the parenchymatous cells increased from 20 μm to 40 μm. These results will be helpful to understand the bud development of ginger seed rhizome during storage.

## 1. Introduction

Ginger (*Zingiber officinale* Roscoe), generally cultivated as the rain-fed crop in Kerala, is planted during April- May, and harvested during December—January. Hence there is a storage period for ginger seed rhizome for 3–4 months from harvest to the next planting season. It is the rest period of a viable seed that fails to germinate even in the presence of favourable environmental conditions. In their native habitat, most genera of the Zingiberaceae family grow well during the rainy season and then go completely dormant during the dry season. Before the onset of dormancy, products of photosynthesis from leaves and pseudo stems will be transferred to accumulate in the rhizome and storage roots [1] leading to rotting, shrinking, sprouting, and rooting resulting in huge losses. Loss upto thirty per cent was reported during storage due to various reasons [2, 3]. To reduce spoilage and to obtain good germination, proper storage of seed rhizome is essential. Zero Energy Cool Chamber (ZECC), is one of the ideal methods to store fresh ginger. The loss in weight of the rhizome was only 23% after storing for four months in ZECC; [4]. The seed rhizome of cultivar Humnabad stored for 90 days under ZECC recorded the highest germination (97.78%) [5]. The ginger rhizomes showed sprouting when stored at room temperature and relative humidity as low as 40 per cent and it also leads to higher weight loss due to the higher respiration rate as a result of sprouting [6]. The sprouting in fresh ginger can be reduced under low-temperature storage (15°C) for up to 4 months [7].

Comparative root anatomy was examined in the rhizomes of *Z*. *officinale* [8] and vascular patterns of secretory structures were histochemically analyzed [5] An extensive study of secretory structures [9] and cellular localization of the important constituents were reported [8]. These studies give information on cell structure. The anatomy of ginger rhizomes on storage has not been studied extensively [4, 10]. Histochemical analysis of 14 species of Zingiberaceae including their secretory structure was reported [11]. But there is no report of the rhizome bud development and histochemical analysis month interval of storage in ZECC in the humid state Kerala, India. Hence the present study was undertaken to elucidate the anatomical morphology and distribution of special cells during bud development in seed rhizomes of ginger (*Z*. *Officinale* Roscoe) under Zero Energy Cool Chamber (ZECC).

## 2. Materials and methods

### 2.1 Plant material storage and experimental design

The seed material of the ginger variety Ashwathy, was collected after the harvest at 210 days after sowing and was stored in the Zero Energy Cool Chambers (ZECC) available in the Department of Plantation Crops and Spices, College of Agriculture, Vellanikkara under Kerala Agriculture University. Seed rhizome parameters and histochemical studies were conducted up to three months from 1^st^ day of storage at monthly intervals. The experiment was laid out in the design CRD with four storage intervals as treatments in three replications.

### 2.2 Observations on seed rhizome

The morphological changes in the seed rhizomes during storage were evaluated. The weight of seed rhizomes stored in the ZECC was recorded at monthly intervals and weight loss was calculated from the mean weight of the sample number of sprouting buds were recorded by counting the newly sprouted buds from randomly selected samples of the stored rhizomes at monthly intervals.


$$Weight\;loss\;(\%)=\frac{\text{Weight}\;\text{of}\;\text{sample}\;\text{after}\;\text{storage}}{\text{Initial}\;\text{trial}\;\text{weight}\;\text{of}\;\text{a}\;\text{sample}}\text{X}\;100$$

### 2.3 Stages of bud development

The stored rhizomes were stained for observing the bud development changes anatomically as described above. The T.S. of rhizome from each sample was analyzed d by using a Trinocular ‘Leica DM 3000’ microscope attached via the ‘Leica DFC 295’ digital camera connected to the computer to know the anatomical changes during storage.

### 2.4 Anatomical characterization of samples

The stored seed rhizomes were examined histochemically to know the changes during storage at monthly intervals. Stages of bud development, number of cork layers, starch grain, and oil globule size were studied.

Hand sections were taken and stained with safranin, washed thoroughly and mounted in 40% glycerin, and observed under the microscope. Trinocular ‘Leica DM 3000’ microscope attached with ‘Leica DFC 295’ digital camera connected to the computer and Leica application suite software was used for the observation and transferring microscopic images of the samples. Images are examined thoroughly and compared to the anatomical characteristics of the rhizome.

To examine the presence of starch grains, a drop of iodine solution (dissolve 2.6 g of iodine and 3 g of potassium iodide insufficient water to produce 100ml) is used which turns blue by indicating the presence of starch. The presence of oil globules was identified by adding Sudan red solution (dissolve 0.5 g of Sudan red in 100 ml of glacial acetic acid (AR) into the specimen which turns orange-pink by indicating the presence of oil globules.

## 3. Results

### 3.1 Observations on seed rhizome

**Fig 1:**
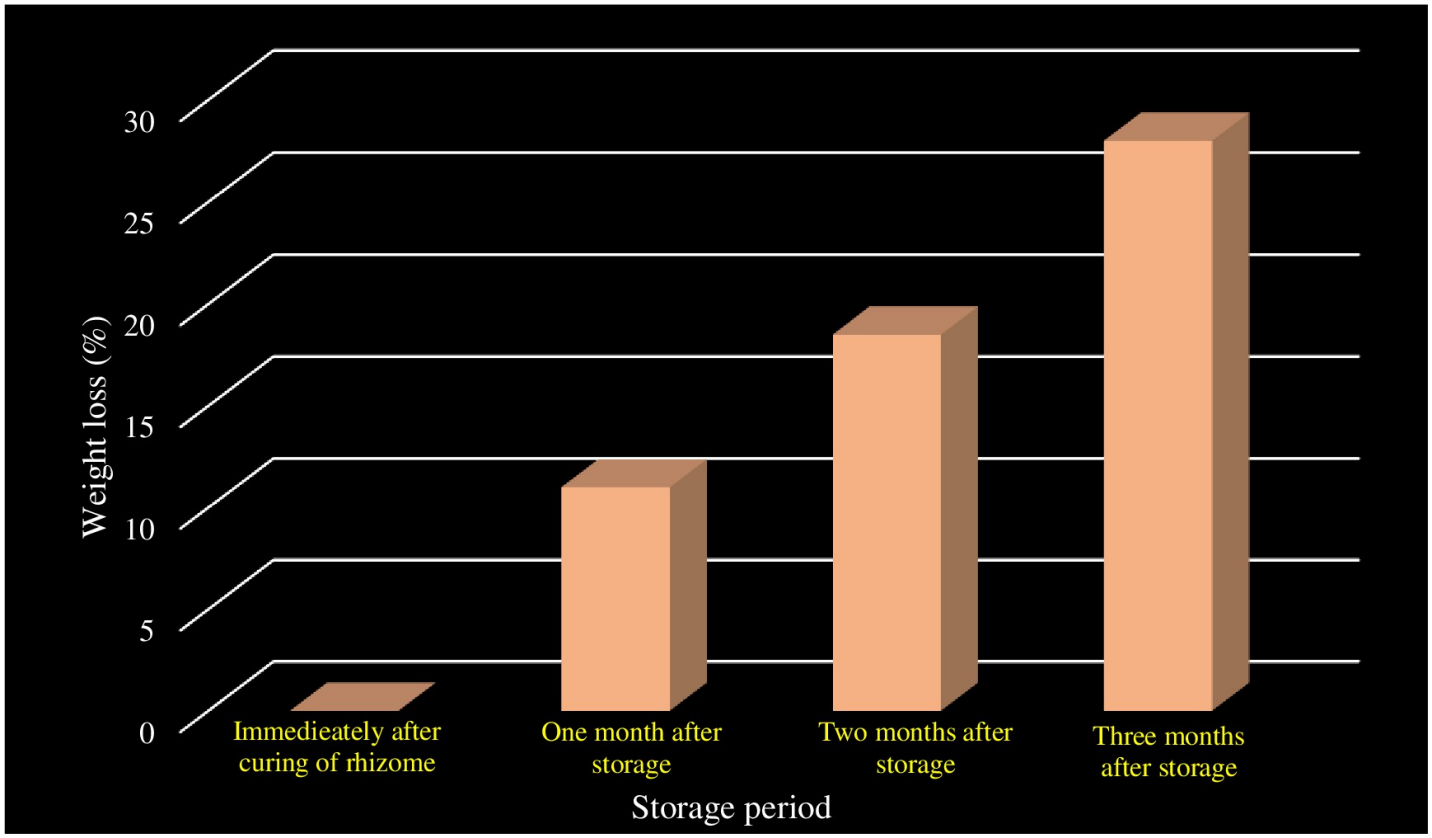
Weight loss of seed rhizome as influenced by storage period.

The morphological changes in the seed rhizomes during ZECC storage were evaluated up to three months from the time of storage at monthly intervals. The weight loss was the highest (28%) at three months after storage (Fig 1). Similarly, the number of buds was maximum (12.25) after three months of storage followed by one month after storage (11.5) and two months after storage (9.75) respectively (Table 1). As the storage period advanced, the weight loss and the number of bud initials increased.

**Table 1:** Effect of storage on weight loss and number of sprouting buds.

Treatments	Weight loss (%)	Number of sprouting buds (per 100g lot)
Immediately after curing of rhizome	0	0
One month after storage	11	9.75
Two months after storage	18.5	11.5
Three months after storage	28	12.25

### 3.2 Stages of bud development

**Fig 2:**
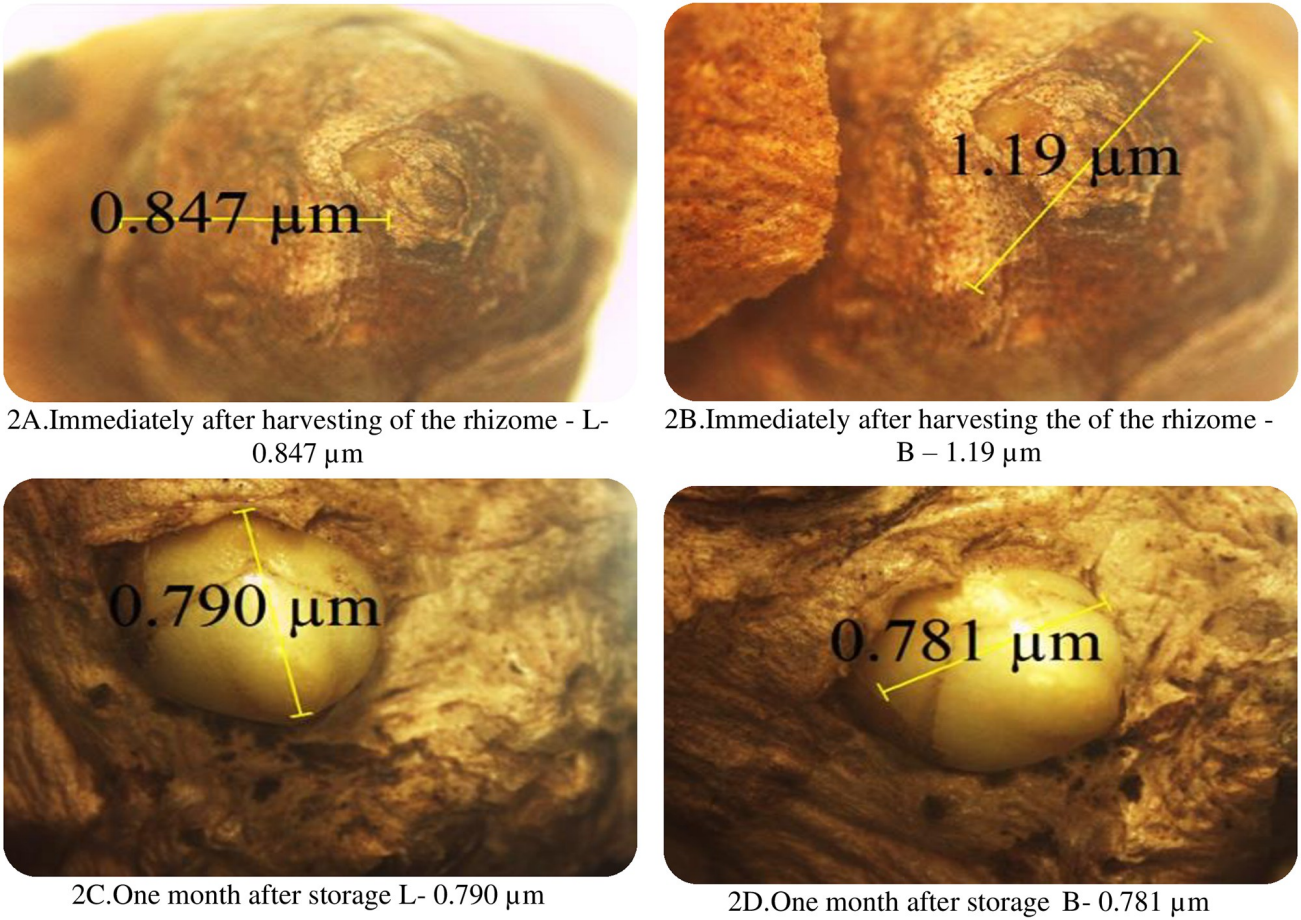
Initial stages of bud development. A. Immediately after harvesting of the rhizome—L- 0.847 μm. B. Immediately after harvesting the of the rhizome—B– 1.19 μm. C. One month after storage L- 0.790 μm. D. One month after storage B- 0.781 μm.

**Fig 3:**
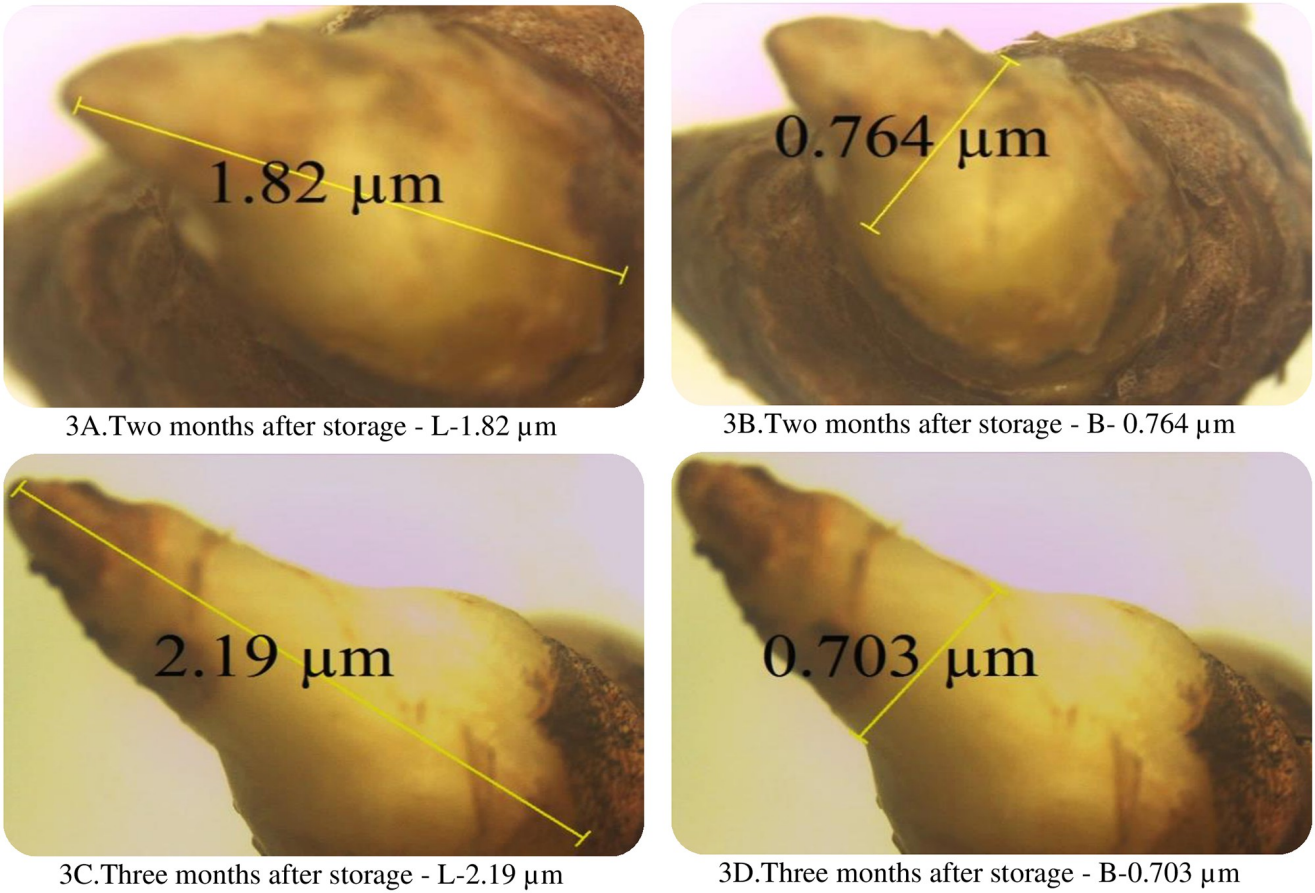
Later stages of bud development. A. Two months after storage—L-1.82 μm. B. Two months after storage—B- 0.764 μm. C. Three months after storage—L-2.19 μm. D. Three months after storage—B-0.703 μm.

The dimensions of the bud in terms of length and breadth showed variation during bud development (Table 2). The length of the bud first reduced and then increased progressively to 2.19 μm (Fig 3C) at three months after storage whereas the breadth reduced from 1.19 μm (Fig 2B) to 0.703μm (Fig 3D) at three months after storage.

**Table 2:** Bud dimensions of the rhizomes as influenced by storage period.

Storage period of Seed Rhizomes	Bud Length (μm)	Bud width (μm)
Fresh seed rhizome	0.847	1.19
One-month-old seed rhizome	0.790	0.781
Two months old seed rhizome	1.82	0.764
Three months old seed rhizome	2.19	0.703

### 3.3 Rhizome—Anatomy

**Fig 4:**
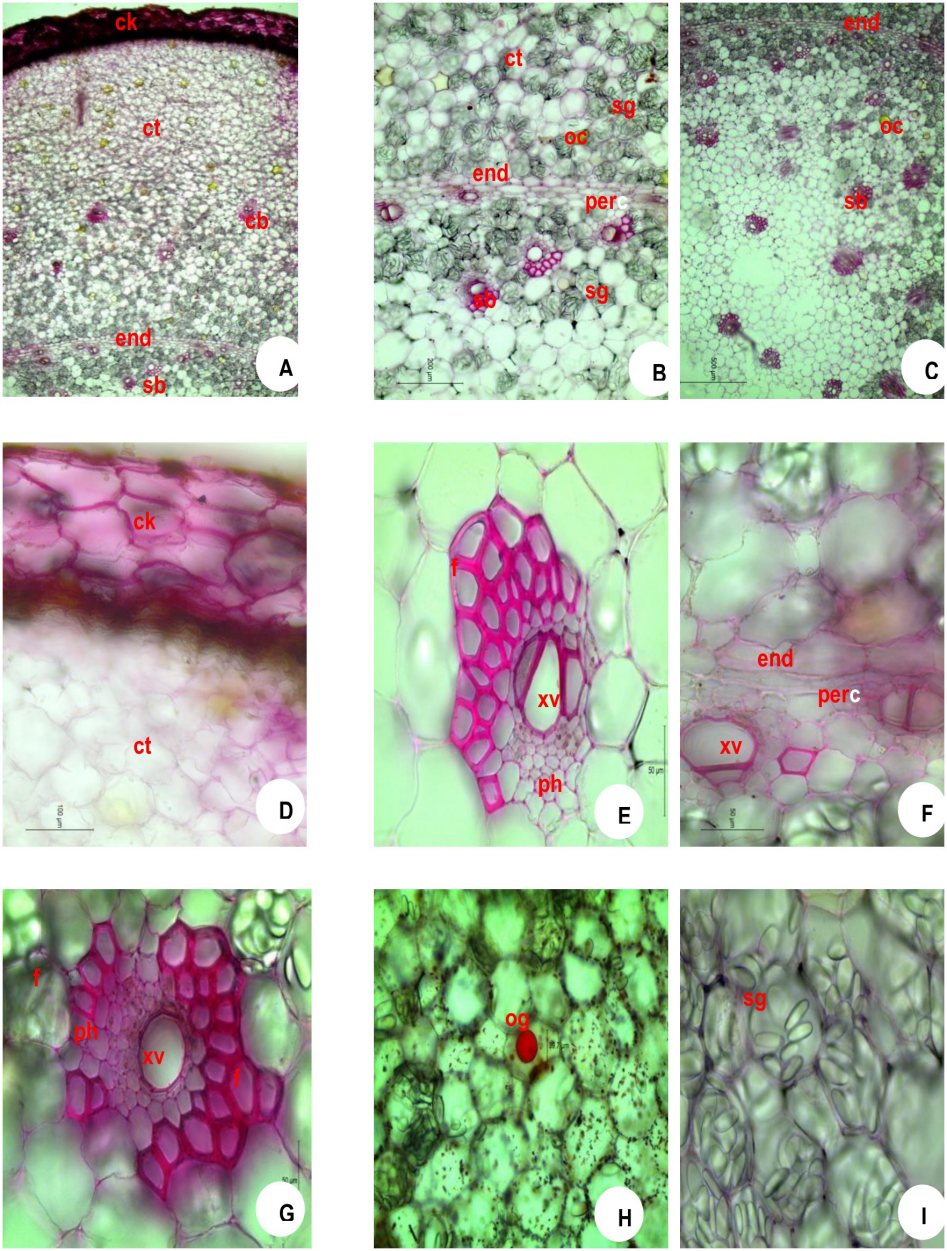
Microscopy of *Zingiber officinale* Rosc. Rhizome (Immediately after harvesting of rhizome). **A**. TS whole; **B**. TS showing endodermal portion; **C**. TS StelaStelarar portion; **D**. cork portion enlarged; **E**. cortical bundle enlarged; **F**. bundles near endodermis enlargStelar Stelar bundle enlarged; **H**. TS showing oil globule; **I**. TS showing starch grains.**CK**, cork; **ct**, cortex; **en**, endodermis; **f**, fibres; **oc**, oleoresin cells; **og**, oil globule; **per**, pericycle; **ph**, phloem; **sb**, Stelar bundle; **sg**, starch grains; **xv**, xylem vessels.

The outermost layer is the epidermal debris often detached at places. Detailed Transverse Section (T.S.) shows 6–15 layers of tangentially elongated thick-walled suberised cork cells (hypodermal cells) which are irregularly arranged. The transverse section of the rhizome shows three distinct zones namely cork (ck), cortex (ct), and stellar region. An outermost thick-walled narrow zone is the cork. It is followed by a wider zone of the parenchymatous cortex which is delimited from the stellar region by the presence of single-layered endodermis (en) (Fig 4A and 4B) and the parenchymatous cells of the entire rhizome are filled with starch grains (sg) and oleoresin cells (oc). The inner cork layers show a somewhat regular arrangement when compared to outer layers (Fig 4D). Below the cork cells, a broader zone of parenchymatous cortical cells can be seen often filled with oblong starch grains and oil globules (og). The cortex (ct) contains numerous collateral closed non-lignified fibro vascular bundles of varying sizes. The vascular bundles of the cortex (ct) and stele form the same structure—xylem vessels (xv) and phloem (ph) elements of fibres (f) at both ends. The bundles lying closer to the pericycle (per) lack fibre (f) covering. (Fig 4E). A layer of elongated endodermis (en) is followed by a narrow pericycle (per) (Fig 4F). Both cortex (ct) and stele contain vascular cortical and stellar bundles (sb) Yellow coloured oil globules (og) are also found in cortical cells (Fig 4H). The starch grains (sg), measuring up to 40 μm and oil globules (og) of 40 μm size can be seen in the parenchyma cells (Fig 4I).

### 3.4 Anatomical observations during storage

**Fig 5:**
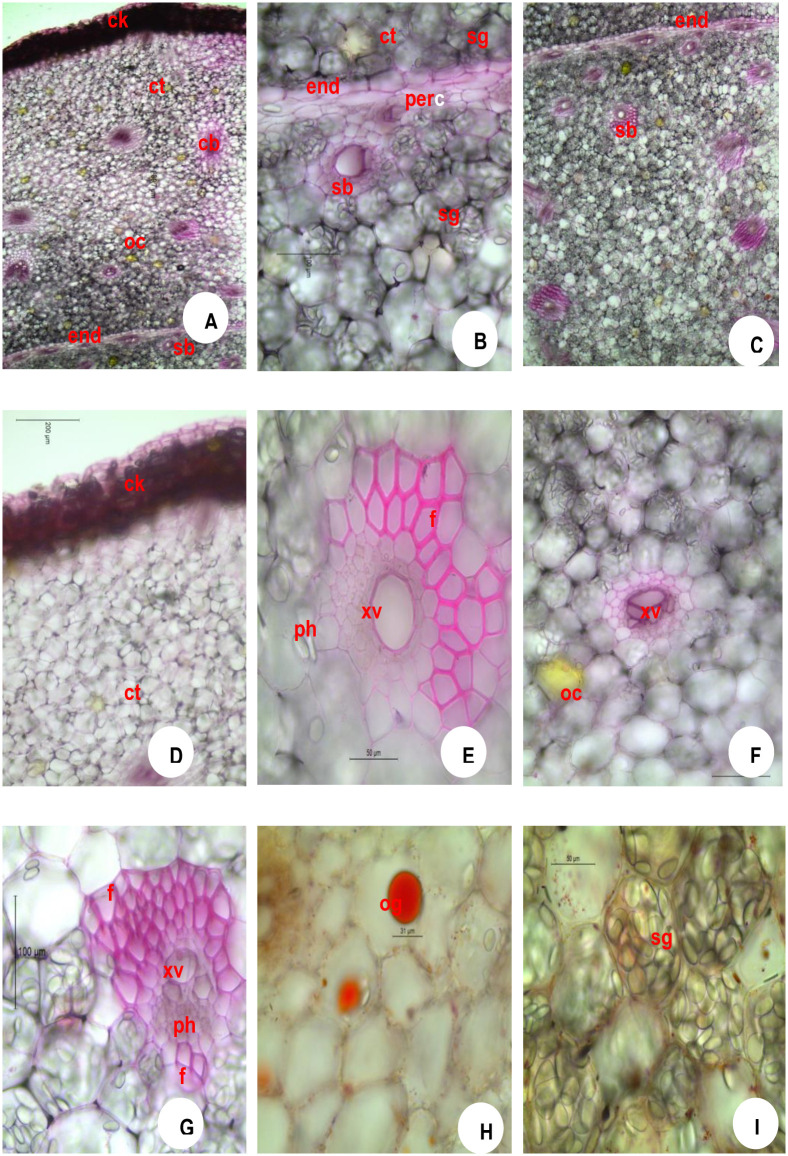
Microscopy of *Zingiber officinale* Rosc. Rhizome (One month after storage). **A**. TS whole; **B**. TS showing endodermal portion; **C**. TS showing Stelar portion; **D**. cork portion enlarged; **E**. cortical bundle enlarged; **F**. bundles near endodermis enlarged; **Stelar**lar bundle enlarged; **H**. TS showing oil globule; **I**. TS showing starch grains.**CK**, cork; **ct**, cortex; **en**, endodermis; **f**, fibres; **oc**, oleoresin cells; **og**, oil globule; **per**, pericycle; **ph**, phloem; **sb**, Stelar bundle; **sg**, starch grains; **xv**, xylem vessels.

**Fig 6:**
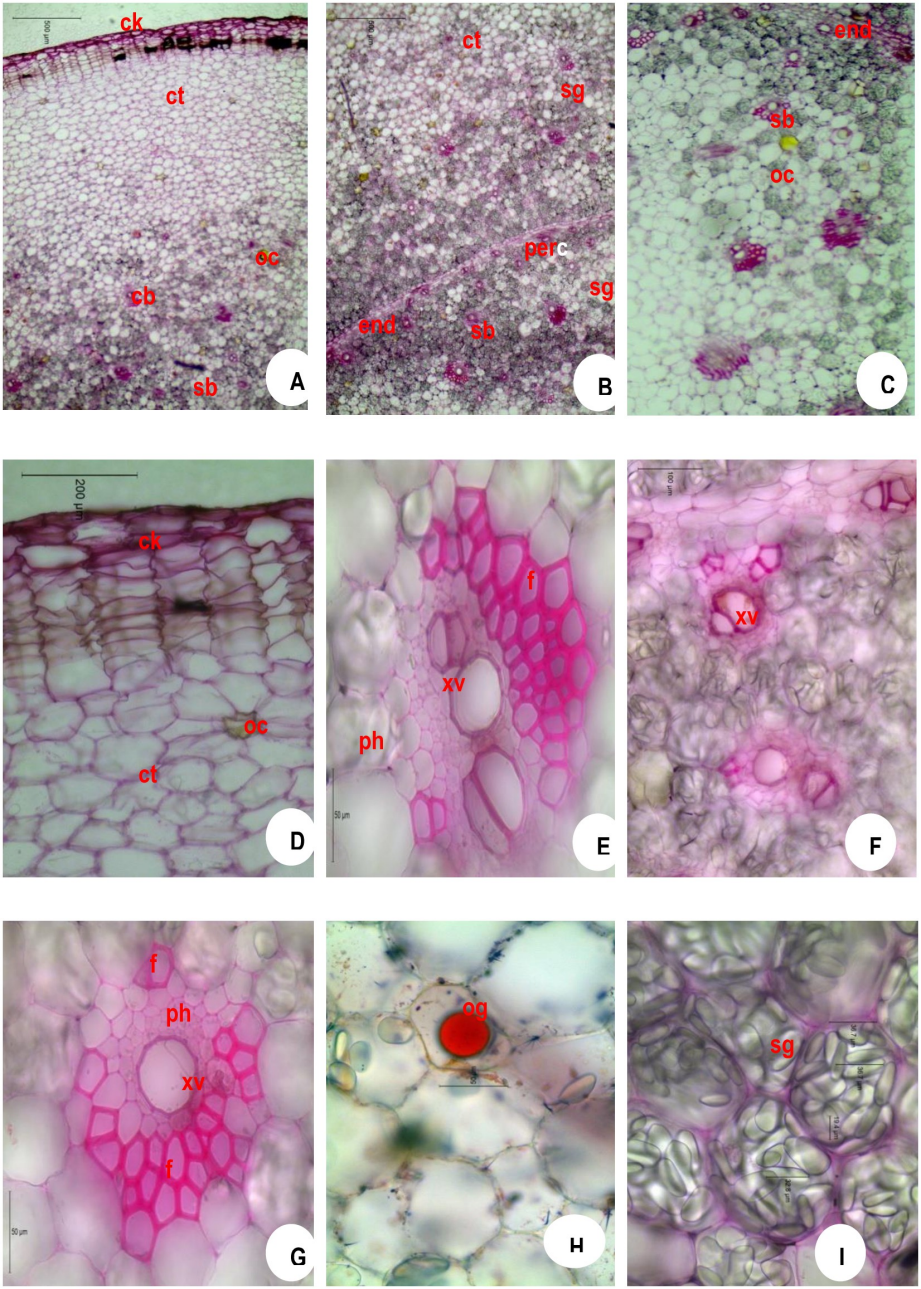
Microscopy of *Zingiber officinale* Rosc. Rhizome (Two months after storage). **A**. TS whole; **B**. TS showing endodermal portion; **C**. TS showing sStelarportion; **D**. cork portion enlarged; **E**. cortical bundle enlarged; **F**. bundles near endodermis enlarged; **Stelar**lar bundle enlarged; **H**. TS showing oil globule; **I**. TS showing starch grains.**CK**, cork; **ct**, cortex; **en**, endodermis; **f**, fibres; **oc**, oleoresin cells; **og**, oil globule; **per**, pericycle; **ph**, phloem; **sb**, sStelarbundle; **sg**, starch grains; **xv**, xylem vessels.

**Fig 7:**
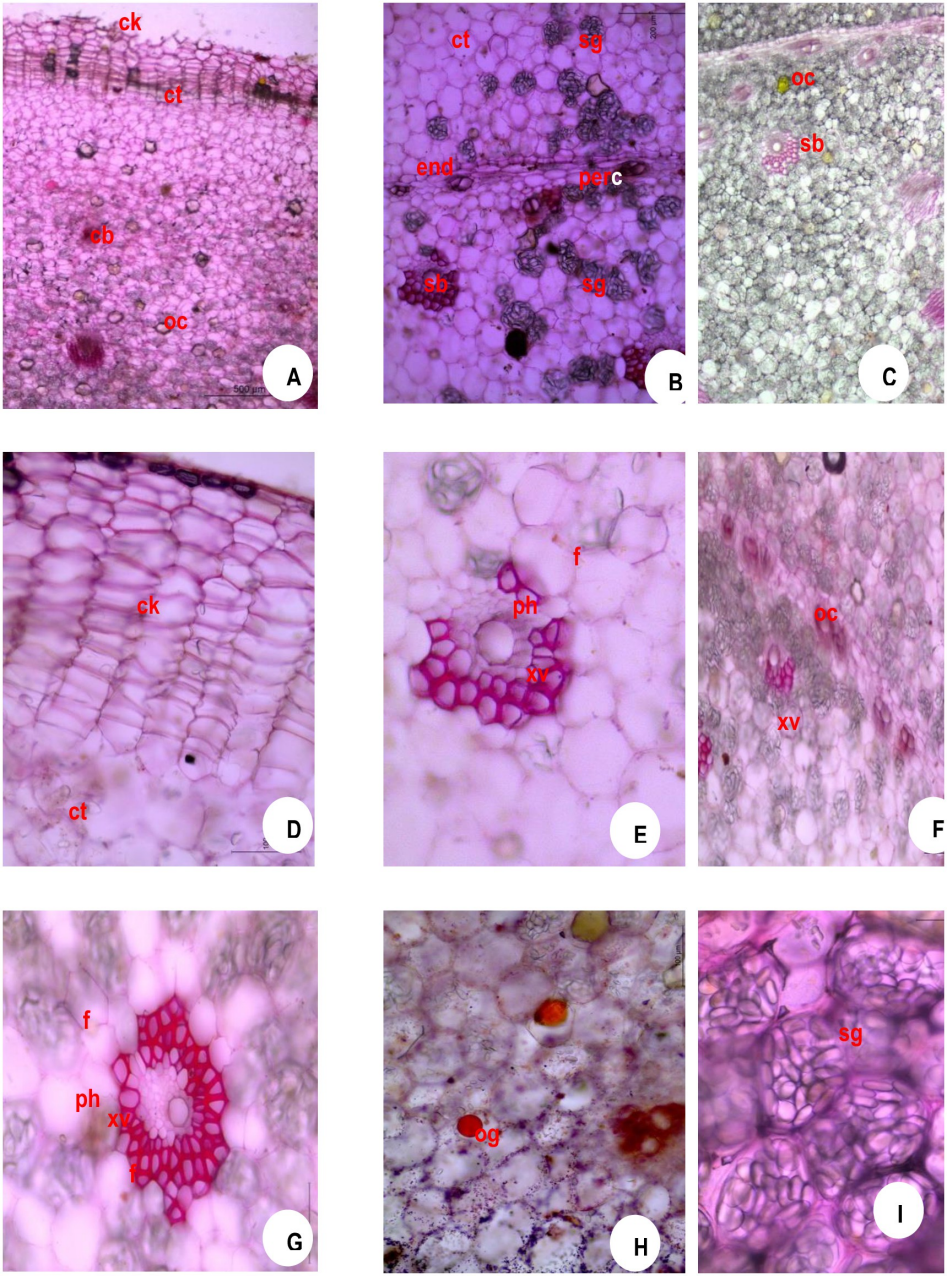
Microscopy of *Zingiber officinale* Rosc. Rhizome (Three months after storage). **A**. TS whole; **B**. TS showing endodermal portion; **C**. TS showingStelarr portion; **D**. cork portion enlarged; **E**. cortical bundle enlarged; **F**. bundles near endodermis enlarged; **GStelar**ar bundle enlarged; **H**. TS showing oil globule; **I**. TS showing starch raincheck, cork; **ct**, cortex; **en**, endodermis; **f**, fibres; **oc**, oleoresin cells; **og**, oil globule; **per**, pericycle; **ph**, phloem; **sb**, Stelar bundle; **sg**, starch grains; **xv**, xylem vessels.

The number of cork layer cells, size of star grains and oil globules are depicted in Table 3. The cork layer cells (hypodermal cells) ranged from 12–15 in number in fresh rhizome seed material of ginger (Fig 4D) which are tangentially elongated in nature. The oil globules (og) were solitary and measuring up to 20 μm in size found inside the parenchymatous cells (Fig 4H) and the starch grains (sg) were oblong measuring up to 40 μm in size and scattered throughout the cortex (ct) and stellar region (Fig 4I). One month after storage, the cork layer cells ranged from 10–15 in (Fig 5D), the oil globules (og) measuring up to 30 μm in size found inside the parenchymatous cells (Fig 5H) and the starch grains were oblong measuring up to 35 μm in size (Fig 5I). Two months after storage, the cork layer cells ranged from 8–15 in number (Fig 6D), the oil globules (og) measuring up to 30 μm in size found inside the parenchymatous cells (Fig 6H) and the starch grains were oblong measuring up to 25 μm in size (Fig 6I). Three months after storage, the cork layer cells ranged from 5–10 in number (Fig 7D), the oil globules (og) were solitary and measuring up to 40 μm in size found inside the parenchymatous cells (Fig 7H) and the starch grains were oblong measuring up to 20 μm in size and scattered throughout the cortex (ct) and stelar region (Fig 7I).

**Table 3:** Anatomical comparison of the rhizomes as influenced by storage period.

Treatments	Number of cork layer cells	Size of Starch grains (μm)	Size of Oil globules (μm)
Fresh seed rhizome	12–15	40	20
One-month-old rhizome	10–15	35	30
Two months old seed rhizome	8–15	25	20
Three months old seed rhizome	5–10	20	40

## 4. Discussion

### 4.1 Observations on seed rhizome

The weight loss observed in the present study supports only 20% reduction of rhizome weight after three months of storage in ZECC [12, 13]. Prior research on turmeric seed rhizomes showed minimum storage losses in the zero-energy chamber [4]. Zero energy cool chamber is an ideal storage structure for ginger and turmeric with enhanced germination, increased plant height, leaf area index, the number of tillers per clump, pseudo stem girth, and fresh rhizome yield [1] The weight loss is mainly due to loss of moisture content and sometimes due to pest infestation [13]. Even maybe due the loss of substrate through respiration in *C*. *longa* [14]. The higher loss in weight was due to the higher respiration rate as a result of sprouting [15].

We confirmed that as the storage period extends, the number of bud initials in the seed rhizome increase in number and it will affect the growth and development of the crop as it acts as the planting material for the next crop. This is in line with, sprouting of ginger during storage is an undesirable character that leads to deterioration [7]. To overcome this, storing the rhizomes under lower temperature (10–15°C) is desirable. Zero Energy Cool Chamber works on the principle of evaporative cooling and maintains high humidity of 95% and reduced temperature (10–15°C) as compared to room temperature. Even the use of ionizing radiations (25 Gy) inhibits sprouting when stored under ambient conditions [1]. In the current study, the rhizomes were desiccated and sprouted and became pale in colour. Decaying of rhizome in ginger rhizomes stored for 3–4 months [16, 17].

### 4.2 Stages of bud development

The length and breadth of the rhizome varied with the storage period. The length of the bud increased width of the bud reduces as the storage period advances. In a rhizome, apex the bud size is proportional to budding bud age, i.e. older buds are larger than younger buds. However, in a rhizome, the size may not be proportional to bud age i.e. younger buds can be larger than older buds. This suggests that some buds in a rhizome branch are subjected to the inhibitory influence [18].

### 4.3 Anatomical observations

The seed rhizomes showed anatomical variations during three months of storage. T.S. of the rhizome zone contains thick-walled narrow zone is the cork. It is followed by a wider zone of the parenchymatous cortex which is delimited from the stellar region by the presence of single-layered endodermis showed a layer of elongated endodermis, followed by a narrow pericycle that induces vascular bundle formation [8, 10, 14, 19–22]. The bundles lying closer to the pericycle (per) fibre (f) covering [23].

We found that T.S of the seed rhizome remains the same throughout the storage period with slight variation [24]. The number of cork layers varies from 10–15. This is a characteristic of the Zingiberaceae which shows an irregular distribution of cork layers [7, 8, 25, 26]. Plesiomorphic type in the Zingiberales is characterized by starch accumulation in the inner part of the cortex and the stele [27]. Initially, the starch grains were larger [12, 28, 29], as the storage period extended, the size of the starch grains reduced as the stored carbohydrate was utilized by the sprouting seed rhizome for its growth. The reduction in starch content is due to the conversion of starch to sugar during storage at all temperatures. This is maybe due to the higher metabolic activities that lead to elongation of the sprouts [7]. In the rhizomes of *C*. *longa* starch content showed a rapid loss (14.07%) from the time of storage till sprouting [17]. The oil globules were found from the initial month and the size of oil globule was found to be increased up to three months of storage [7, 30–33]. Histological examination of ginger peel confirms decreasing levels of essential oil during rhizome development [34, 35]. The size of oil cells is dependent on their number, Oil globule number decreases as the size of globules increases [14].

## 5. Conclusion

In the tropical humid climatic situation of Kerala, storing the seed rhizomes in a zero-energy cool chamber showed a minimum physiological loss in weight. This method of storage acts as an alternative method to the other traditional storage methods to conserve valuable seed material. The bud enhancement in terms of length and breadth showed the physiological chart that acts as a positive way to overcome the dormancy period. Our study highlights the anatomical particularly in the number of cork layers, increase in the size of the oil globule and reduction in starch grains and the composition of secret structure at various intervals of storage that ultimately helps the behavioral nature of the ginger rhizome.

## Supporting information

S1 File (DOCX)

## References

[1] Shadap A, Hegade N K, Lyngodh Y A, Rymbai H. Effect of storage methods and seed rhizome treatment on field performance of ginger var. Humnabad. Indian Journal of Hill farming. 2014; 27(1):219–228.

[2] Melati S H, Palupi E R, Susila A D. Growth, yield and quality of ginger from produced through early senescence. International Journal of Applied Science and Technology. 2016; 6(1): 21–28.

[3] Panneerselvam R, Jaleel C A, Somasundaram R, Sridharan R, Gomathinayagam M. Carbohydrate metabolism in *Dioscorea esculenta* (Lour.) Burk. tubers and *Curcuma longa* L. rhizomes during two phases of dormancy. Colloids Surf. B: Biointerfaces. 2007; (59): 59–66. doi: 10.1016/j.colsurfb.2007.04.006 17531451

[4] Kirankumar G. Studies on storage of turmeric seed rhizomes. M.Sc. (Ag.) thesis, University of Agricultural Sciences, Dharwad, India. 2001; 98p.

[5] Borah P. Saikia M. Studies on characterization of starch granules from botanicals using foldscope microscopy. Progressive Horticulture. 2020; 52(1).

[6] Yusof N. Sprout inhibition by gamma irradiation in fresh ginger (*Zingiber officinale* Roscoe). Journal of Food Processing and Preservation. 1990; 14:113–122.

[7] Uma E, Muthukumar T. Comparative root morphological anatomy of Zingiberaceae. Systematics and Biodiversity. 2014; 12(2):195–209

[8] Tomlinson P B. Studies on the systematic anatomy of the Zingiberaceae. Botanical Journal of the Linnean. Society. 1956; (55): 547–92.

[9] Mu N, Liu H F, Kuang Y F, Zou P, Liao J P. Developmental processes of rhizome and ultrastructure of secretory cavities in *Zingiber officinale* Roscoe. Journal of Tropical and Subtropical Botany. 2015; 23(2):151–159.

[10] Zarate R Yeoman M M. Studies of the cellular localization of the phenolic pungent principle of ginger, *Zingiber officinale* Roscoe. The New Phytologist. 1994; 126:295–300.

[11] Liu H, Specht CD, Zhao T, Liao J. Morphological Anatomy of Leaf and Rhizome in *Zingiber officinale* Roscoe, with Emphasis on Secretory Structures. Hortscience. 2020; 55(2):204–207.

[12] Sharma Y. Ginger (*Zingiber officinale*)-An elixir of life a review. The Pharma Innovation Journal 2017; 6(10): 22–27.

[13] Indriyani S. Secretory structure and histochemistry test of some Zingiberaceae plants. AIP Conference Proceedings 2017; 1908:040008, 10.1063/1.5012722

[14] Paull R E, Chen N J, Goo T T C. Compositional changes in ginger rhizomes during storage. American Society of Horticultural. Science. 1988;13:584–588.

[15] Remashree A B, Unnikrishnan K, Ravindran P N. Development of oil cells and ducts in ginger (*Zingiber officinale* Roscoe). Journal of Spices and Aromatic Crops. 1999; 8 (2): 163–170.

[16] Haines C P. Insects and arachnids of tropical stored products: their biology and identification. 2nd. edn. (a training manual). Natural Resources Institute (NRI), Chatham Maritime, UK. 1991.

[17] Phongpreecha K. Unpublished data. Chiangrai Horticultural Research Center, Thailand. 1997; 126p.

[18] Shukor A R, Aziz I A, Shokri O A. Physical changes of fresh ginger rhizomes as influenced by storage temperature and duration. MARDI Research Bull. 1986; 4(3): 243–248.

[19] Rai S, Hossain M. Comparative studies of three traditional methods of seed rhizome storage of ginger (*Zingiber officinale* Roscoe) practised in Sikkim and Darjeeling Hills. Environmental Ecology. 1998(16):34–36.

[20] Shah J J, Raju E C. General morphology, growth, and branching behaviour of the rhizomes of ginger, turmeric, and mango ginger. New Bot. 1975;11(2): 59–69.

[21] Abdulrahman A A, Taiwo M O, Oladle F A, Phytopharmaceutical potential and microscopic analysis of rhizomes of Curcuma longa and *Zingiber officinale* (Zingiberaceae). Annals of West University of Timişoara, ser. Biology, 2015; (2), pp. 73–86.

[22] Chinese Pharmacopoeia Commission. Pharmacopoeia of the People’s Republic of China. China Medical Science Press, Beijing. 2015.

[23] de Menezes NL, Silva D C, Arruda R C, Melo-de-Pinna G F, Cardoso V A, Castro N M, et al. The meristematic activity of the endodermis and the pericycle in the primary thickening in monocotyledons: Considerations on the ‘‘PTM”. Annals of the Brazilian Acadamy of Sciences. 2005; 77(2):259–274. 10.1590/s0001-37652005000200006 15895162

[24] Unnikrishnan K, Ravindran P N. Sherlija K K. Bud and root development of turmeric (Curcuma longa L.) rhizome Journal of Spices and Aromatic Crops. 1999; 8 (1): 49–55.

[25] Das A, Pal K K, Nag S. Anatomy, micromorphology and histochemical localization of different phytochemicals of two medicinally important taxa of the family Zingiberaceae Research. Journal of Life Science. Informatics Publications. 2018; 4(1):19–198p.

[26] Eltahir AS, Elnoor MI, Menahi S, Mustafa EMA. Morph-Anatomical Studies and Anti-bacterial Activities of the Rhizome of *Zingiber officinale* Roscoe. Open Access Library Journal. 2018; 5: 4890.

[27] Gracie A J, Brown P H, Burgess S W, Clark R J. Rhizome dormancy and shoot growth in myoga (*Zingiber miogaa* Roscoe). Scientia Horticulturae 2000; 84(1–2):27–36.

[28] Chomicki G. Analysis of rhizome morphology of the Zingiberales in Payamino (Ecuador) reveals convergent evolution of two distinct architectural strategies, Acta Botanica Gallica, 2013; 160:3–4, 239–254.

[29] Anderson T, du Plessis S F, Niem TR. Evaporative cooling of ginger (*Zingiber officinale*). Acta Horticulture 1990; 275:173–179.

[30] Nambiar M C. Morphological and cytological investigations in the genus Curcuma L. PhD (Ag.) thesis, University of Bombay, Mumbai, India. 1979.

[31] Ghosh N, Veloz M, Sherali N, Das A, Chatterjee A, Bennett J, et al. Microscopic characterization of some medicinal plants and elemental analysis of Triphala (three fruits) with anticarcinogenic properties. Journal of Medicinal Herbs and Ethnomedicine 2016; 2(2): 11–18

[32] Paz M D P P. Rhizome manipulation affects growth and development of ornamental gingers PhD thesis. Department of Horticulture, Louisiana State University. 2003; 100p.

[33] Zhan KY, Yin H Z, Zhang X Z, Xu K. Chemical composition analysis on SEF CO2 extracts of seed ginger and fresh ginger. Journal of Food Science. 2009; 30:33–35.

[34] Manglakumari C K, Ninan C A, Mathew A G. Histochemical studies on the constituents of ginger. Journal of Plantation Crops. 1984; 12: 146–151.

[35] Oluwaseyi OS, Oyekunle OJ, Babatola L A. Effects of pre-storage treatments and storage methods on sprouting and nutritional quality of ginger (*Zingiber officinale* Rosc) rhizomes. Direct Research Journal of Agriculture and Food Science. 2017; 5 (4): 207–213.

