# Supplementary material for: Histochemical analysis and storage behaviour of Ginger (Zingiber officinale Roscoe) under Zero-Energy Cool Chamber (ZECC)
Source: PLoS One. 2022 May 5;17(5):e0265320. doi: 10.1371/journal.pone.0265320 (PMC9070942; doi:10.1371/journal.pone.0265320)
Supplement: S1 File — (DOCX) [file pone.0265320.s001.docx]

**Anatomical characterization of samples**

**Equipments Used**

**Microscopes**:

**Normal compound microscope with camera attachment**:

Trinocular ‘Leica DM 3000’ microscope attached with ‘Leica DFC 295’ digital camera connected to the computer and Leica Application Suite software was used for the observation and transferring microscopic images of the samples.

**Stains used**

**Saffranin** – Dissolve 1g saffranin in 100ml distilled water.

**Anatomical characterization of samples**

Hand sections were taken and stained with diluted aqueous saffranin, washed thoroughly and mounted in 40% glycerin and observed under the microscope. anatomical microphotographs were transferred using the computer controlled microscopic system and camera. Images are examined thoroughly and compared the anatomical characteristics.

#### **Table 1. Effect of storage on weight loss and number of sprouting buds**

| **Treatments** | **Weight loss (%)** | | | **Number of sprouting buds**  **(per 100g lot)** | | |
| --- | --- | --- | --- | --- | --- | --- |
|  | **R_1_** | **R_2_** | **R_3_** | **R_1_** | **R_2_** | **R_3_** |
| Immediately after curing of rhizome | 0 | 0 | 0 | 0 | 0 | 0 |
| One month after storage | 10 | 9 | 14 | 12.5 | 9 | 7.75 |
| Two months after storage | 19 | 16 | 20.5 | 9 | 12.5 | 13 |
| Three months after storage | 27 | 25.5 | 31.5 | 11.5 | 10 | 15.25 |

#### **Table 3. Anatomical comparison of the rhizomes as influenced by storage period**

| **Treatments** | **Number of cork layer cells** | | | **Size of Starch grains (**µm**)** | | | **Size of Oil globules (**µm**)** | | |
| --- | --- | --- | --- | --- | --- | --- | --- | --- | --- |
|  | **R_1_** | **R_2_** | **R_3_** | **R_1_** | **R_2_** | **R_3_** | **R_1_** | **R_2_** | **R_3_** |
| Fresh seed rhizome | 13 | 12 | 15 | 35 | 40 | 45 | 10 | 20 | 30 |
| One-month-old rhizome | 11 | 15 | 13 | 40 | 35 | 30 | 25 | 35 | 30 |
| Two months old seed rhizome | 15 | 8 | 12 | 20 | 30 | 25 | 15 | 25 | 20 |
| Three months old seed rhizome | 7 | 10 | 5 | 15 | 20 | 25 | 40 | 45 | 35 |
